# Supplementary material for: The Alteration of CTNNBIP1 in Lung Cancer
Source: Int J Mol Sci. 2019 Nov 13;20(22):5684. doi: 10.3390/ijms20225684 (PMC6888110; doi:10.3390/ijms20225684)
Supplement: Supplementary file 1 [file ijms-20-05684-s001.pdf]

Table S1. Correlation between mRNA Expression and DNA Methylation *of CTNNBIP1* gene in Lung Cancer Patients [a].

			CTNNBIP1 mRNA [b]			
Characteristics			Total	− (%)	+	*p* value [d]
Overall			121	61 (50.4)	60	
CTNNBIP1 methylation probes (region) [c]	cg02833588 (5'UTR)	U	62	16 (25.8)	46	<0.001
		M	59	45 (76.3)	14	
	cg17953816 (5'UTR)	U	67	28 (41.8)	39	0.035
		M	54	33 (61.1)	21	
	cg24086140 (5'UTR)	U	65	23 (35.4)	42	<0.001
		M	56	38 (67.9)	18	

[a] These results were analyzed in data from the lung cancer patients of the publicly available data in GSE66836 and GSE66863 projects by the GEO database. [b] CTNNBIP1 mRNA value < mean indicates "−". [c] CTNNBIP1 methylation probes value > mean+1SD indicates "M". M, hypermethylation; U, unmethylation. [d] The *p* value for each analysis is provided by $\chi2$ analysis.

Table S2. List of primer sequences used in the present study.

Gene	Primer	5′ 3′ Sequences	PCR Size (bp)	Tm (°C)	Cycle Number
CTNNBIP1	Forward<br>Reverse	GAGCACCTGTTTGCCTGAAG<br>GCCCTTCAACAGCATCCAGG	321	60	35
MMP7	Forward<br>Reverse	TACAGTGGGAACAGGCTCAGG<br>GGCACTCCACATCTGGGCT	199	62	35
cyclin D1	Forward<br>Reverse	ATGTGTGCAGAAGGAGGTCC<br>CTTAGAGGCCACGAACATGC	199	60	35
c-MYC	Forward<br>Reverse	TGAAAGGCTCTCCTTGCAGC<br>GCTGGTAGAAGTTCTCCTCC	175	60	35
GAPDH	Forward<br>Reverse	AATCCCATCACCATCTTCCA<br>CCTGCTTCACCACCTTCTTG	588	55	30
β-actin	Forward<br>Reverse	GGCGGCACCACCATGTACCCT<br>AGG GGCCGGACTCGTCATACT	180	60	35
CTNNBIP1-methyl-M	Forward<br>Reverse	GTTTAACGACGTAGGAAGATTACGT<br>ATTTAAAAACGAAAACAACAAAACG	129	55	35
CTNNBIP1-methyl-U	Forward	AGTTTAATGATGTAGGAAGATTATG	125	55	35

Reverse	AAAACAAAAACAACAAAACAAA

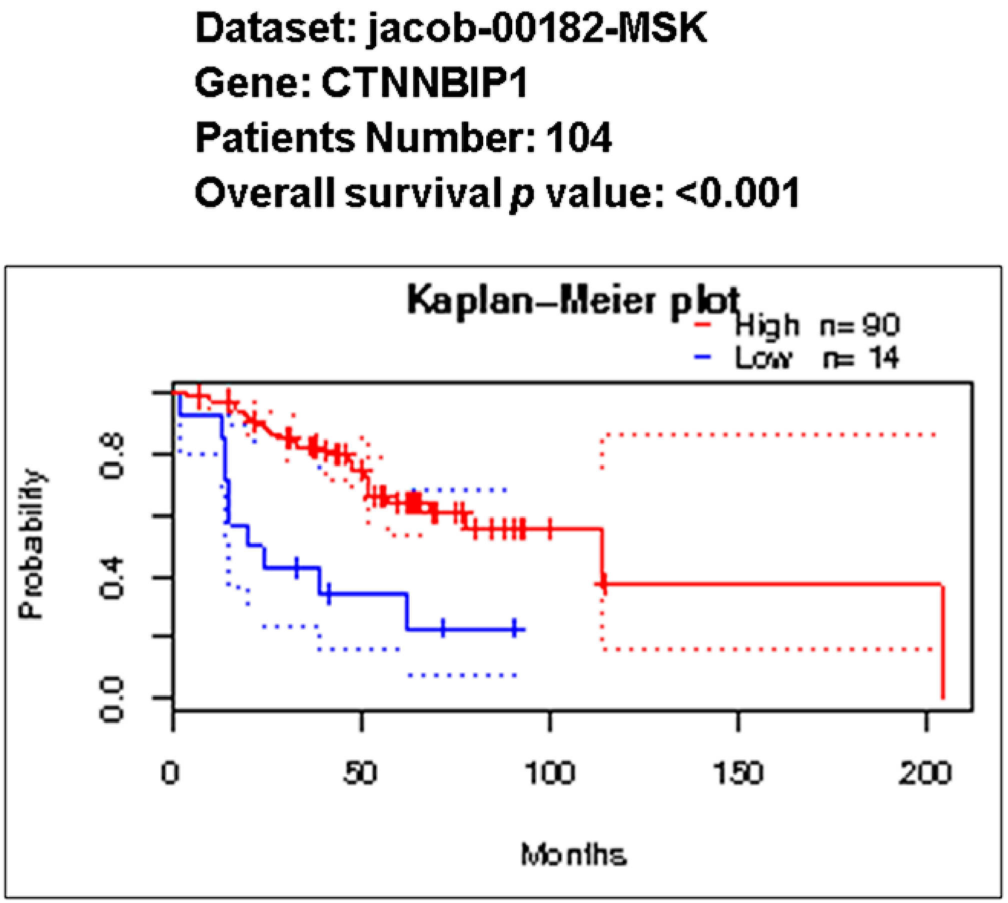

Figure S1. The relationship between the expression of CTNNBIP1 and patient prognosis of lung cancer patients in jacob-00182-MSK project.

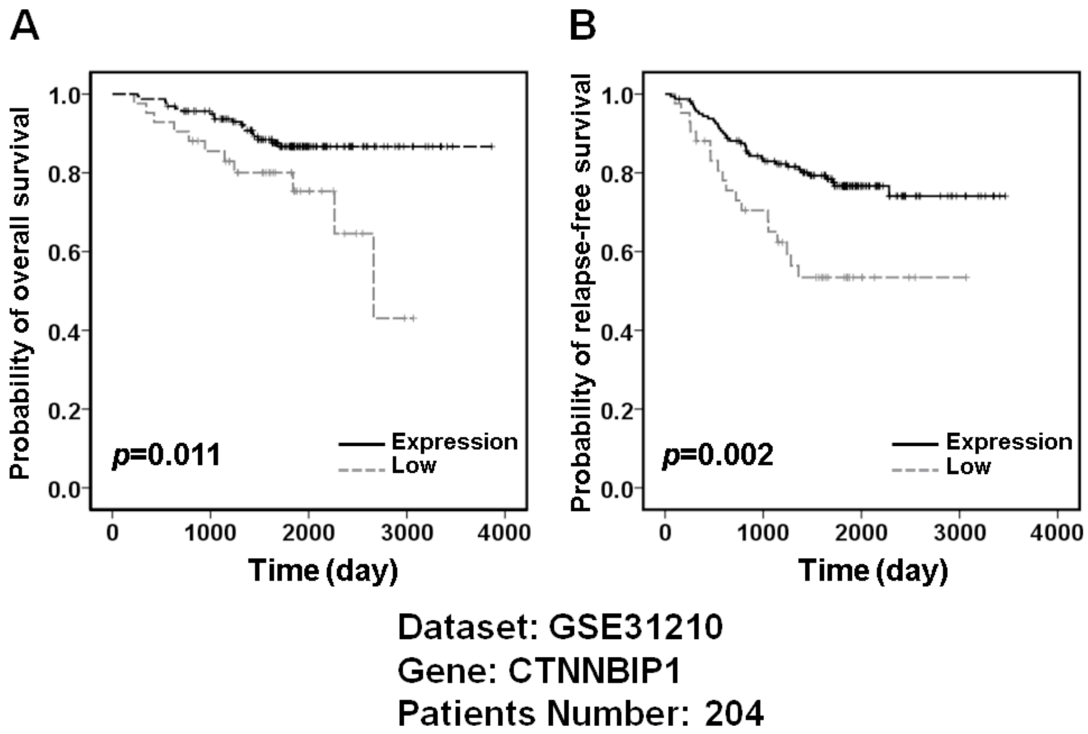

Figure S2. Low CTNNBIP1 expression correlates with poor survival of lung cancer patients in GSE31210 project.
